# Radiofrequency ablation for DYT‐28 dystonia: short term follow‐up of three adult cases

**DOI:** 10.1002/acn3.51170

**Published:** 2020-09-04

**Authors:** Shiro Horisawa, Kenkou Azuma, Hiroyuki Akagawa, Taku Nonaka, Takakazu Kawamata, Takaomi Taira

**Affiliations:** ^1^ Department of Neurosurgery Tokyo Women’s Medical University Tokyo Japan; ^2^ Tokyo Women’s Medical University Institute for Integrated Medical Sciences Tokyo Japan

## Abstract

Mutations in the lysine methyltransferase 2B (*KMT2B*) gene have recently been reported to be associated with childhood‐onset generalized dystonia. There have been no studies investigating ablative treatments for the management of this disorder. Three patients underwent either a staged unilateral pallidotomy and contralateral pallidothalamic tractotomy (19‐year‐old man, 2‐year follow‐up), a unilateral pallidothalamic tractotomy (34‐year‐old man, 6‐month follow‐up) or a simultaneous unilateral pallidothalamic tractotomy and ventro‐oral thalamotomy (29‐year‐old man, 6‐month follow‐up). The average total patient score on the Burke‐Fahn‐Marsden Dystonia Rating Scale‐Movement Scale improved from 39.5 to 13.2 (66.6%) after the procedures. No significant complications were identified. Ablative treatments appear to be a promising alternative surgical option for generalized dystonia with KMT2B mutation.

## Introduction

Mutations in the lysine methyltransferase 2B (*KMT2B*) gene have been recently reported to be associated with childhood‐onset generalized dystonia (DYT‐28 dystonia), a movement disorder that is characterized by limb onset and progression to cranio‐cervico‐axial involvement.^1^, ^2^ Other phenotypic features of this disorder include mild cognitive decline, short stature, and microcephaly. Due to the limited number of reports about DYT‐28, the genetic contribution and the clinical course of its dystonic features still remain unknown.

Several studies have shown significant improvements in patients with DYT‐28 after deep brain stimulation (DBS) of the globus pallidus internus (GPi).^3^, ^4^, ^5^, ^6^, ^7^; however, the use of ablative treatments for DYT‐28 management have not yet been reported. For patients who develop dystonia at a young age, ablative treatments may play a particularly valuable role in disease management because they do not necessitate device implantation or time‐consuming and costly postoperative management. Here, we report surgical outcomes of radiofrequency ablations performed in three patients with DYT‐28 generalized dystonia and *KMT2B* mutations.

## Case Reports

The ethics committee of the Tokyo Women’s Medical University approved the study. Written informed consent was obtained from all patients.

Detailed patient characteristics and surgical outcomes are shown in Tables 1 and 2, respectively.

**Table 1:** Clinical characteristics.

Case	Sex	Age at onset (years)	KMT2B variant			Family history	Microcephaly	Short statue	Cognitive impairment	Initial symptoms	Sites affected
			Variation nucleotide	Variation amino acid	Variant type						
1	Male	6	c.5631delG	p.G1879Vfs*15	Frameshift	none	Yes	Yes	Mild	Right foot inversion	Larynx, Neck, Bilateral arm, Trunk, Bilateral leg
2	Male	10	c.C2275T	p.Q759*	Nonsense	none	Yes	Yes	Mild	Bilateral arms stiffness	Larynx, Neck, Bilateral arm, Trunk
3	Male	14	c.2422_2423insCAG	p.QdelinsPE	Inframe insertion	none	Yes	Yes	None	Right hand stiffness	Larynx, Neck, Right arm, Trunk

KMT2B, lysine methyltransferase 2B.

**Table 2:** Surgical outcomes.

		Case 1			Case 2		Case 3	
		Pre	6 months^1^	24 months^2^	Pre	6 months	Pre	6 months
BFM‐MS	Total	66	34	10.5	32	21	20.5	8
	Eyes							
	Mouth							
	Speech/Swallow	3	2	2			1	1
	Neck	4	2	0.5	8	6	4.5	3
	Rt Arm	12	0	0	6	6	9	0
	Lt Arm	16	16	4	6	0		
	Trunk	12	4	2	12	9	6	4
	Rt Leg	12	2	1				
	Lt Leg	8	8	2				

Abbreviations: BFM‐MS, Burke‐Fahn‐Marsden Dystonia Rating Scale‐Movement Scale.

^1^ Scores after left pallidotomy.

^2^ Scores after right pallidothalamic tractotomy and left pallidotomy.

### Patient 1

**Figure 1:**
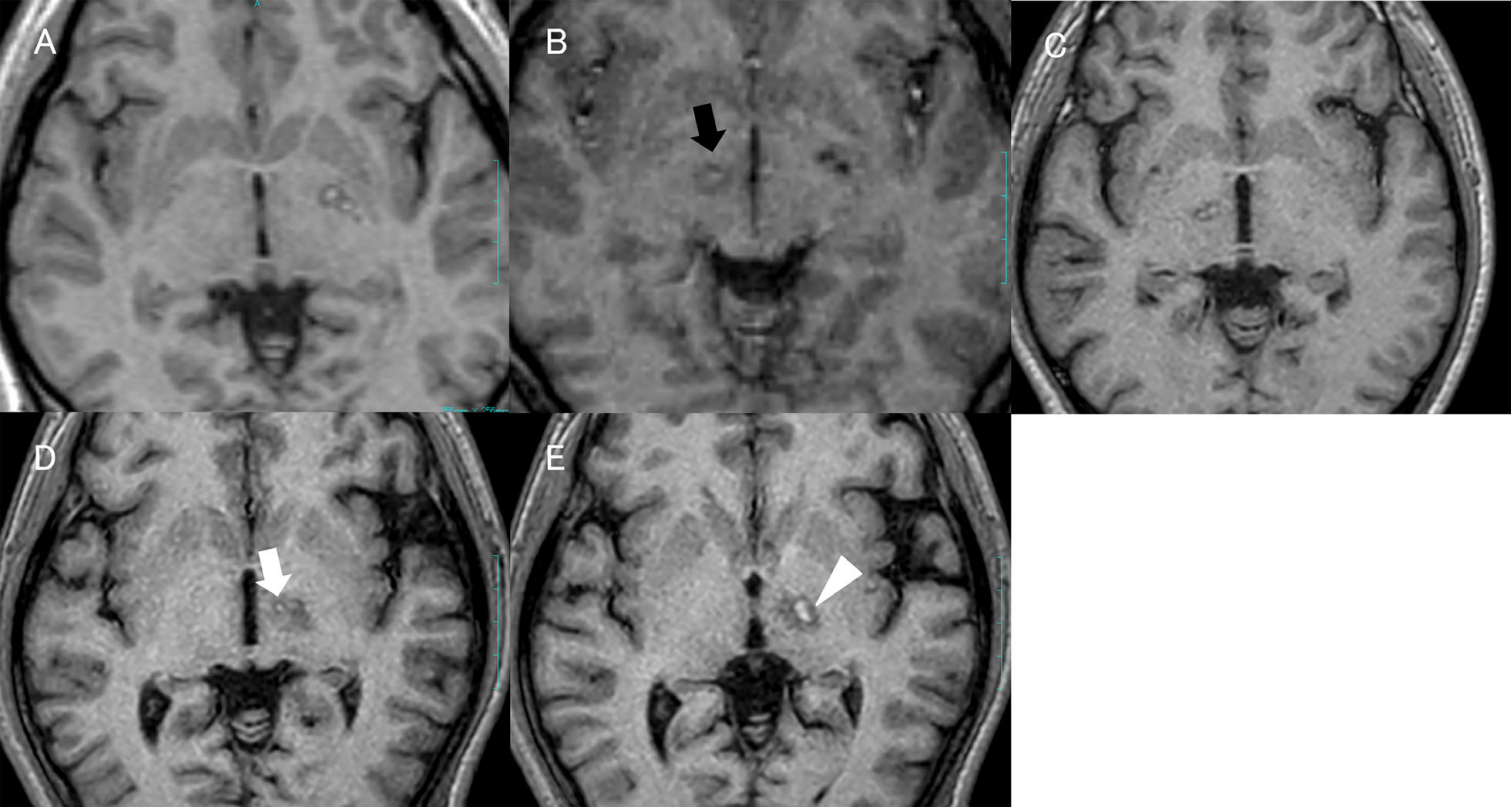
T1‐weighted magnetic resonance imaging on the day of surgery. Coagulated lesions on left globus pallidus internus (A) and right pallidothalamic tract (B, black arrow) of Patient No. 1, (C) Right pallidothalamic tract of Patient No. 2, (D) Left pallidothalamic tract (arrow) of Patient No. 3, and (E) Left ventro‐oral nucleus of the thalamus (arrowhead) of Patient No. 3.

Patient No. 1 was a 19‐year‐old man with generalized dystonia and a confirmed *KMT2B* mutation. His development slowly decelerated after birth, resulting in microcephaly, short stature, cognitive decline, and dysarthria. At 6 years of age, left foot inversion developed. Two years later, myoclonic movements in bilateral arms and truncal bending developed. At 15 years of age, his truncal bending worsened, resulting in wheelchair dependence, and dysphonic symptoms developed. His disease progressed with time, and treatment with several oral medications and botulinum toxin injections was ineffective. As a result, he was referred to our hospital for surgical treatment. At presentation, his most significant symptom was truncal bending while walking or standing. His preoperative Burke‐Fahn‐Marsden Dystonia Rating Scale‐Movement Scale (BFM‐MS) score was 66 (Video S1). Because of his predominantly right‐sided hand myoclonus, the patient underwent a left pallidotomy, which resulted in significant improvement of the right hand myoclonus (Figure 1A). His trunk bending, however, was not improved (Video S1). Six months after the left pallidotomy, a right pallidothalamic tractotomy was performed. After this procedure, his trunk bending and left hand myoclonus were both significantly improved. However, the independent walking was still unsteady due to residual dystonic movements of left leg and trunk until 1‐month post‐operative evaluation. These symptoms were gradually improved and the patient began walking independently (BFM‐MS 10) (Video S1). At the 2‐year follow‐up, his dystonic symptoms were stable and no significant postoperative complications were found. For detailed information about the pallidotomy and pallidothalamic tractotomy procedures, please refer to our previous reports.^8^, ^9^

### Patient 2

Patient No. 2 was a 34‐year‐old man with generalized dystonia and a *KMT2B* mutation. Bilateral jerky hand movements initially developed at 10 years of age. These jerky movements remained stable for approximately 20 years, and the patient was employed without significant difficulty. At 32 years of age, however, the patient developed neck and trunk tilting to the left, resulting in unemployment. Oral medications, including anticholinergics and clonazepam, as well as botulinum toxin injections, were not effective. After 1 year, his axial symptoms suddenly deteriorated, and he was referred to our hospital for surgical treatment. His preoperative BFM‐MS score was 32. Due to his predominantly left‐sided dystonic symptoms (Video S2), the patient underwent a right pallidothalamic tractotomy, which resulted in significant improvement of his left hand myoclonic movements, but only slight improvements in his trunk and neck bending (Figure 1B, Video S2). At the 6‐month follow‐up, his axial symptoms were still disabling (BFM‐MS: 21), and he was scheduled to undergo surgery on the contralateral side. There was no significant postoperative complications.

### Patient 3

Patient No. 3 was a 29‐year‐old right‐handed man with generalized dystonia and a *KMT2B* mutation. At 14 years of age, he developed difficulty in writing with his right hand and progressive difficulty in extending his right elbow. He was subsequently diagnosed with dystonia. Treatment with anticholinergics, levodopa, clonazepam, and botulinum toxin injections were attempted, without significant symptomatic improvement. Twelve years after onset, neck tilting, trunk bending, and right shoulder elevation developed (Video S3), and he was referred to our hospital for surgical treatment. His preoperative BFM‐MS was 20.5. Due to his predominantly right‐sided symptoms, the patient underwent a simultaneous left ventro‐oral thalamotomy and a left pallidothalamic tractotomy (Figure 1B). After the procedures, his right hand symptoms had completely improved; however, his axial symptoms did not show significant improvement. While 6‐month clinical follow‐up, there was no gradual improvement of axial dystonic symptoms. No significant complications were identified. His postoperative BFM‐MS at the 6‐month follow‐up was 7.

## Discussion

This report is the first to describe the use of ablative surgeries in patients with DYT‐28 dystonia, and these cases demonstrate three important findings. First, limb symptoms responded very well to contralateral ablations. Second, unilateral ablations were not sufficient to successfully improve axial symptoms. Third, bilateral ablations greatly improved axial dystonic symptoms, as previous studies using GPi‐DBS have also reported.

Various mutations in the *KMT2B* gene have been reported to be associated with the development of generalized DYT‐28 dystonia.^1^, ^2^, ^5^, ^7^, ^10^, ^11^, ^12^, ^13^, ^14^, ^15^, ^16^, ^17^ Detailed characteristics of the three cases reported here are shown in Table 1. We did not perform genetic testing in their parents. However, the parents of three patients were all normal without any movement or developmental abnormalities. Therefore, three patients were expected to have de novo mutations in KMT2B gene. All three cases had myoclonic jerks in the upper limbs that responded very well to contralateral ablations. The average contralateral side of limbs score on the BFM‐MS greatly improved after treatment, from 10.5 ± 3.6 preoperatively to 1.2 ± 1.6 at final follow‐up (88.6% improvement). Axial symptoms, however, responded insufficiently after unilateral ablations (neck: 32.7%, trunk: 30% improvement). Only Patient No. 1 underwent a staged bilateral ablation, with significant improvement of axial symptoms (neck: 87.5%, trunk: 83.3%). This outcome suggests that bilateral interventions may be required to successfully improve axial dystonic symptoms. Dystonia in the larynx region was found to be less likely to respond to ablative treatments (25% improvement). No significant complications were identified in these patients.

Previous studies have shown significant improvements in patients with DYT‐28 dystonia after bilateral GPi‐DBS, with 5 to 10 years of improvement after the initial procedure.^1^, ^6^ However, the majority of these studies did not document outcomes using objective evaluation scales, such as the BFM rating scale or the Toronto Western Spasmodic Torticollis Scale. Only five reports documented the efficacy of GPi‐DBS for DYT‐28 dystonia using the BFMDRS.^3^, ^4^, ^5^, ^6^, ^7^ Kawarai et al. used the BFMDRS to report post‐GPi‐DBS outcomes in three patients with DYT‐28, showing 70.5%, 45.7%, and 31.1% improvements in BFMDRS values.^7^ Dafsari et al. reported the longest follow‐up period (10 years) of a single patient who underwent GPi‐DBS and was found to have a 34.9% improvement in BFMDRS values.^6^ Mun et al. reported an 83.3% improvement in BFMDRS scores after bilateral GPi‐DBS at 22‐month follow‐up.^3^ In the present study, there was an 84.1% improvement in the BFM‐MS score after bilateral ablation and a 34.4% and 61.0% improvement in the BFM‐MS score after unilateral ablations, which corresponded to previous results using GPi‐DBS. Dystonia in the larynx region has previously been reported to be less likely to respond to GPi‐DBS, which is also consistent with our results.^5^, ^7^ Kawarai et al. reported that two out of three patients with DYT‐28 dystonia who underwent GPi‐DBS did not show improvement in their dysphonia.^7^ Only one case report showed significant improvement (87.5%) in speech and swallowing aspects of the BFMDRS at 7‐month follow‐up.^4^

Patients who develop generalized dystonia at a young age are likely to require neurosurgical treatments, including DBS or ablative procedures. The reported mean age of onset for DYT‐28 is 6 years, and disease progression to generalized dystonia generally requires 2 to 10 years.^18^ This pattern suggests that patients who undergo DBS may require postoperative management for 50 years or more. During this timeframe, the DBS implant may develop hardware‐related complications, including infection, rejection, or device malfunctions.^19^ Additionally, a large number of patients may not be able to afford treatment with DBS or may not be able to access it due to geographic reasons. Ablative treatments have several significant advantages, including the absence of hardware‐related complications and the avoidance of significant postoperative management needs. In addition, the medical expenses associated with ablative treatments are much lower than those of DBS. However, ablative treatments could provide irreversible side effects such as hemiparesis, dysarthria, visual field deficit or dysesthesia by injuring surrounding structures. Therefore, careful observation for adverse effects respond to macrostimulation through treatment electrode before making permanent lesions. In this study, there was no significant postoperative complications in three patients.

The safety of bilateral ablative treatment has not yet been elucidated, and bilateral pallidotomies may lead to unpredictable and poor outcomes, including parkinsonism and delayed cerebral infarctions. Therefore, we utilized a staged one‐sided pallidotomy and then contralateral pallidothalamic tractotomy in patients requiring bilateral ablative surgeries for dystonia. We have recently reported the preliminary outcomes of this strategy.^8^ The long‐term effects of ablative treatments in patients with hereditary dystonia, who have a high likelihood of disease progression over time, have not yet been confirmed. By lacking of adjustability and reversibility, ablative treatments may not be insufficient when the disease progresses. Larger sample sizes, longer follow‐up periods, and more extensive evaluations are necessary to confirm the safety of bilateral ablative surgeries.

The present study demonstrated that these three patients with DYT‐28 dystonia responded well to ablative treatments, which suggests that ablative treatments appear to be a promising alternative surgical option for generalized dystonia with KMT2B mutation. Due to the limitation of this study including small number of patients and short follow‐up period, further studies with larger sample sizes and longer follow‐up period are needed to confirm the safety and long‐term efficacy of ablative treatments for DYT‐28 dystonia.

## Conflict of Interest

The authors declare that the research was conducted in the absence of any commercial or financial relationships that could be construed as a potential conflict of interest.

## Author Contributions

SH acquired and analyzed the data and drafted the manuscript. KA and HA collected and analyzed the data. TN acquired the data. TK supervised this study. TT acquired and analyzed the data and supervised this study.

## Supporting information


**Video S1**. Pre‐ and post‐operative condition after surgery in Case 1

**Video S2**. Pre‐ and post‐operative condition after surgery in Case 2.

**Video S3**. Pre‐ and post‐operative condition after surgery in Case 3.
